# Supplementary material for: Using Moderate Transgene Expression to Improve the Genetic Sexing System of the Australian Sheep Blow Fly Lucilia cuprina
Source: Insects. 2020 Nov 13;11(11):797. doi: 10.3390/insects11110797 (PMC7697711; doi:10.3390/insects11110797)
Supplement: Supplementary file 1 [file insects-11-00797-s001.pdf]

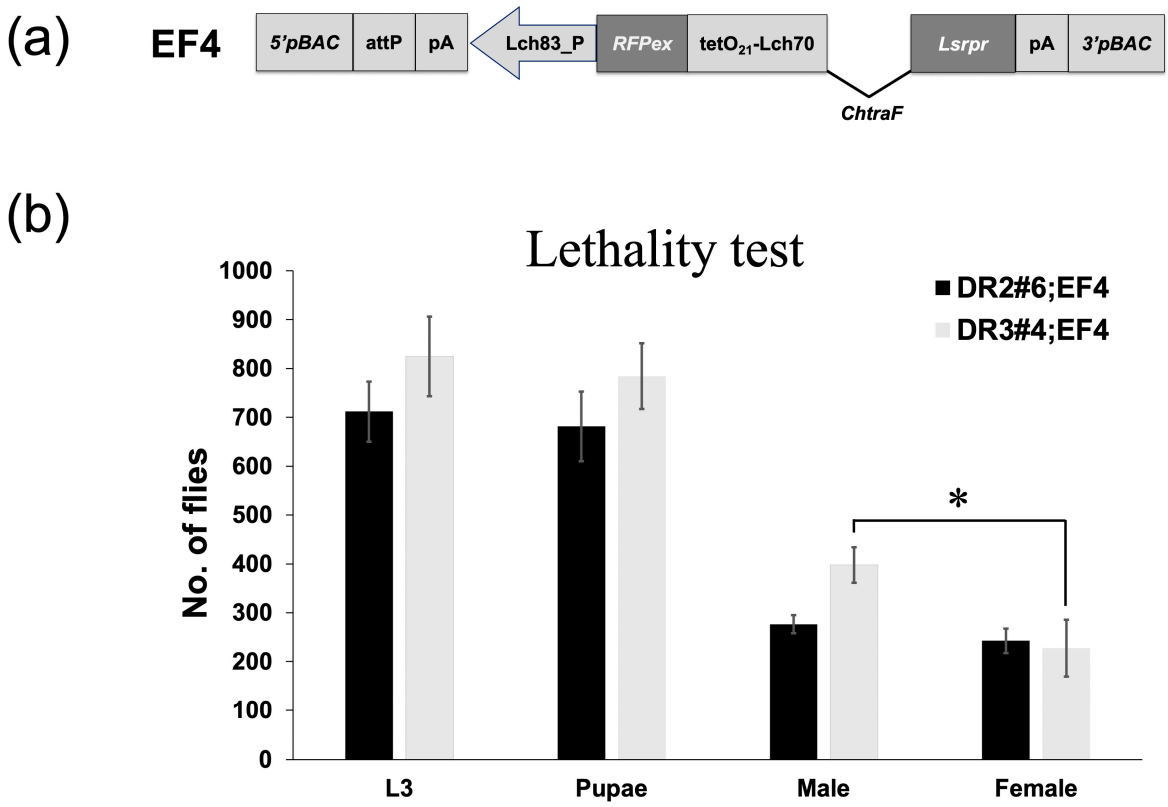

Figure S1. Female viability of TESSs with a *L. sericata reaper* effector gene. (**a**), the EF4 gene construct contains a marker cassette (*Lchsp83* promoter-RFPex-Lctub polyA) and a sex-specific effector cassette ($tetO_{21}$-*Lchsp70* core promoter-*Chtra* female-specific intron-*Lsrpr*-SV40 polyA) flanked by the ends of the *piggyBac* transposon. (**b**), male and female viability of the DR2#6;EF4 and DR3#4;EF4 DH strains raised on diet without tetracycline. Eggs collected from 10 pairs of adults from each DH strain and the insects reared on 93% beef. The mean number of third instar larvae (L3), pupae and adult male and female with standard error are shown ($n = 3$). In the previous study, seven independent EF1 lines were obtained and all of them achieved 100% female lethality when crossed with DR2#6 at double heterozygous condition, and seven independent EF3 lines were obtained and six of them achieved 96–100% female lethality (the remaining one showed 66% female lethality) when tested under the same condition [16]. In addition, DR3#4 also achieved high female lethality when tested with several EF1/EF3 lines at double heterozygous condition, and relatively high *tTA* expression was identified in the ovaries from DR3#4 [17]. Here DR2#6;EF4 showed no female lethality at double homozygous condition, indicating less pro-apoptotic activity of *Lsrpr* compared to that of *Lshid* in *L. cuprina*. On the other hand, 64% female lethality was detected from DR3#4;EF4 DH strain, possibly due to the relatively high tTA expression at late developmental stages from DR3#4 (Figure 1), suggesting the killing efficiency of *Lsrpr* effector also depends on tTA expression from the driver. Therefore, both the expression of the driver and effector are important for female lethality in the absence of tetracycline.
